# *Leishmania infantum* infection modulates messenger RNA, microRNA and long non-coding RNA expression in human neutrophils *in vitro*

**DOI:** 10.1371/journal.pntd.0012318

**Published:** 2024-07-19

**Authors:** Natália Francisco Scaramele, Jéssica Antonini Troiano, Juliana de Souza Felix, Sidnei Ferro Costa, Mariana Cordeiro Almeida, Flávia Regina Florencio de Athayde, Matheus Fujimura Soares, Maria Fernanda da Silva Lopes, Amanda de Oliveira Furlan, Valéria Marçal Felix de Lima, Flavia Lombardi Lopes

**Affiliations:** 1 Department of Production and Animal Health, São Paulo State University (Unesp), School of Veterinary Medicine, Araçatuba, São Paulo, Brazil; 2 Department of Animal Clinic, Surgery and Reproduction, São Paulo State University (Unesp), School of Veterinary Medicine, Araçatuba, São Paulo, Brazil; Bernhard Nocht Institute for Tropical Medicine, Hamburg, Germany, GERMANY

## Abstract

In the Americas, *L*. *infantum* (syn. *chagasi*) is the main cause of human visceral leishmaniasis. The role of neutrophils as part of the innate response to *Leishmania* spp. infection is dubious and varies according to the species causing the infection. Global expression of coding RNAs, microRNAs and long non-coding RNAs changes as part of the immune response against pathogens. Changes in mRNA and non-coding RNA expression resulting from infection by *Leishmania* spp. are widely studied in macrophages, but scarce in neutrophils, the first cell to encounter the trypanosomatid, especially following infection by *L*. *infantum*. Herein, we aimed to understand the expression patterns of coding and non-coding transcripts during acute *in vitro* infection of human neutrophils by *L*. *infantum*. We isolated neutrophils from whole blood of healthy male donors (n = 5) and split into groups: 1) infected with *L*. *infantum* (MOI = 5:1), and 2) uninfected controls. After 3 hours of exposure of infected group to promastigotes of *L*. *infantum*, followed by 17 hours of incubation, total RNA was extracted and total RNA-Seq and miRNA microarray were performed. A total of 212 genes were differentially expressed in neutrophils following RNA-Seq analysis (log_2_(FC)±0.58, FDR≤0.05). *In vitro* infection with *L*. *infantum* upregulated the expression of 197 and reduced the expression of 92 miRNAs in human neutrophils (FC±2, FDR≤0.01). Lastly, 5 downregulated genes were classified as lncRNA, and of the 10 upregulated genes, there was only 1 lncRNA. Further bioinformatic analysis indicated that changes in the transcriptome and microtranscriptome of neutrophils, following *in vitro* infection with *L*. *infantum*, may impair phagocytosis, apoptosis and decrease nitric oxide production. Our work sheds light on several mechanisms used by *L*. *infantum* to control neutrophil-mediated immune response and identifies several targets for future functional studies, aiming at the development of preventive or curative treatments for this prevalent zoonosis.

## Author summary

Visceral leishmaniasis is a neglected tropical disease that causes fever, weight loss, anemia and swelling of liver and spleen. About 2500 cases are reported annually in the Americas, with a high mortality rate. Understanding how the immune system of people with visceral leishmaniasis responds to this parasite is essential for the development of preventive and curative methods. In order to understand how gene expression is modulated during visceral leishmaniasis, we infected *in vitro* cultured human neutrophils, the first immune cells to be recruited in this infection, with *Leishmania infantum*, the protozoan that causes visceral leishmaniasis in the Americas. Next, we measured the expression of coding RNAs, responsible for the production of proteins required for an effective immune response, and of non-coding RNAs, able to control these coding RNAs, thus helping or hindering host response to infection. Analysis of coding and non-coding RNAs points to an attempt by the parasite to modulate the transcriptome of host cells, influencing the host’s response to infection. Our work identifies several targets for future functional studies, aiming at the development of preventive or curative treatments for this prevalent zoonosis.

## Introduction

*Leishmania* genus cause the neglected tropical disease leishmaniasis, some *Leishmania* spp. cause cutaneous injury (e.g. *L*. *major*, *L*. *mexicana*, *L*. *amazonensis*, *L*. *braziliensis*) while other species can cause damage to internal organs resulting in visceral leishmaniasis (VL) (e.g. *L*. *donovani*, *L*. *infantum*) [1]. In the Americas, *L*. *infantum* (syn. *chagasi*) is the main cause of VL in mammals, especially humans (69,665 cases from 2001 to 2021) [2]. In 2021, 93.5% of human VL cases reported in the American continent occurred in Brazil, leading to the highest mortality rate since 2012 [2].

Immune response is paramount to the establishment of defense against infections [3]. In innate immunity, macrophages are already established as the main *Leishmania* host cells and [4], neutrophils also have known functions in the early stages of *Leishmania* infection, such as the formation of neutrophil extracellular traps and cytokine production [5]. Notwithstanding, authors suggest that neutrophils serve as shelter for *Leishmania* prior to the infection of macrophages [6–8], and that *Leishmania* is able to attenuate neutrophil action [9], while others defend the importance of neutrophil recruitment and its protective role [10]. It is consensus that neutrophil action seems to vary according to the infecting *Leishmania* species [11]. Whether this duality susceptibility *vs*. resistance in neutrophils is due to transcriptome and epigenetic changes remains to be elucidated.

Changes in global expression of messenger RNAs (mRNAs) can shed light on response to metabolic disorders, i.e. obesity [12], autoimmunity, cancer immunity, as well as immune response against a plethora of pathogens [reviewed [13,14]]. Transcriptomic studies were conducted in dogs, mice, and humans infected with several *Leishmania* species [15–20]. In 2022 Maruyama *et al*. [21] used whole blood mRNA-Seq analysis to investigate co-expression between mRNA and long non-coding RNAs (lncRNAs) during *L*. *infantum* natural infection and, most recently, Fernandes *et al*. [22] investigated the influence of three *Leishmania* species, including *L*. *infantum*, on lncRNA and mRNA expression in human macrophages.

LncRNAs are a type of non-coding RNA (ncRNA) that, despite its low abundance and poor conservation among species [23], are known to regulate numerous physiological and pathological processes, including host response to infectious diseases [24,25]. Its action can be better defined depending on its location within a cell, presence in the nucleus would indicate direct transcriptional control over DNA, whilst presence in the cytoplasm could indicate post-transcriptional action via binding to mRNAs [25], or small ncRNAs, named microRNAs (miRNAs).

MiRNAs are a short sequence (~22 nucleotides) ncRNA with post-transcriptional regulatory function [26,27], mediated by binding of the seed region (6~8 first nucleotides) of a mature miRNA [28] to the 3’ UTR end of mRNAs, blocking translation, or breaking complementary mRNAs [29]. LncRNAs may bind with the seed regions of miRNAs and reduce the availability of these miRNAs, in yet another ncRNA regulatory process termed competitive-endogenous RNA [30]. Also, miRNA expression is temporally regulated, and varies in different tissues [31], and has already been implicated in host immune response [32–35]. In response to VL-causing species (*L*. *donovani* and *L*. *infantum*), miRNAs have been shown to be modulated in different hosts [36–41], and seem to regulate important immune mechanisms [42,43]. Literature also suggests that miRNAs produced by *Leishmania* may influence its pathogenicity [44].

Changes in mRNA and ncRNA expression occurring post *Leishmania* spp. infection are widely studied in macrophages, as previously reviewed [45,46]; however, studies are scarce regarding *Leishmania* infection in neutrophils [47], and absent in this cell type when we target *L*. *infantum* infection, the most relevant species for VL in the Americas. Thus, our aim was to analyze the co-expression of coding and non-coding transcripts in an acute infection of neutrophils by *L*. *infantum*, employing an *in vitro* model of acute neutrophilic response to this prevalent infection.

## Results

### *L*. *infantum* infection of human neutrophils

**Fig 1:**
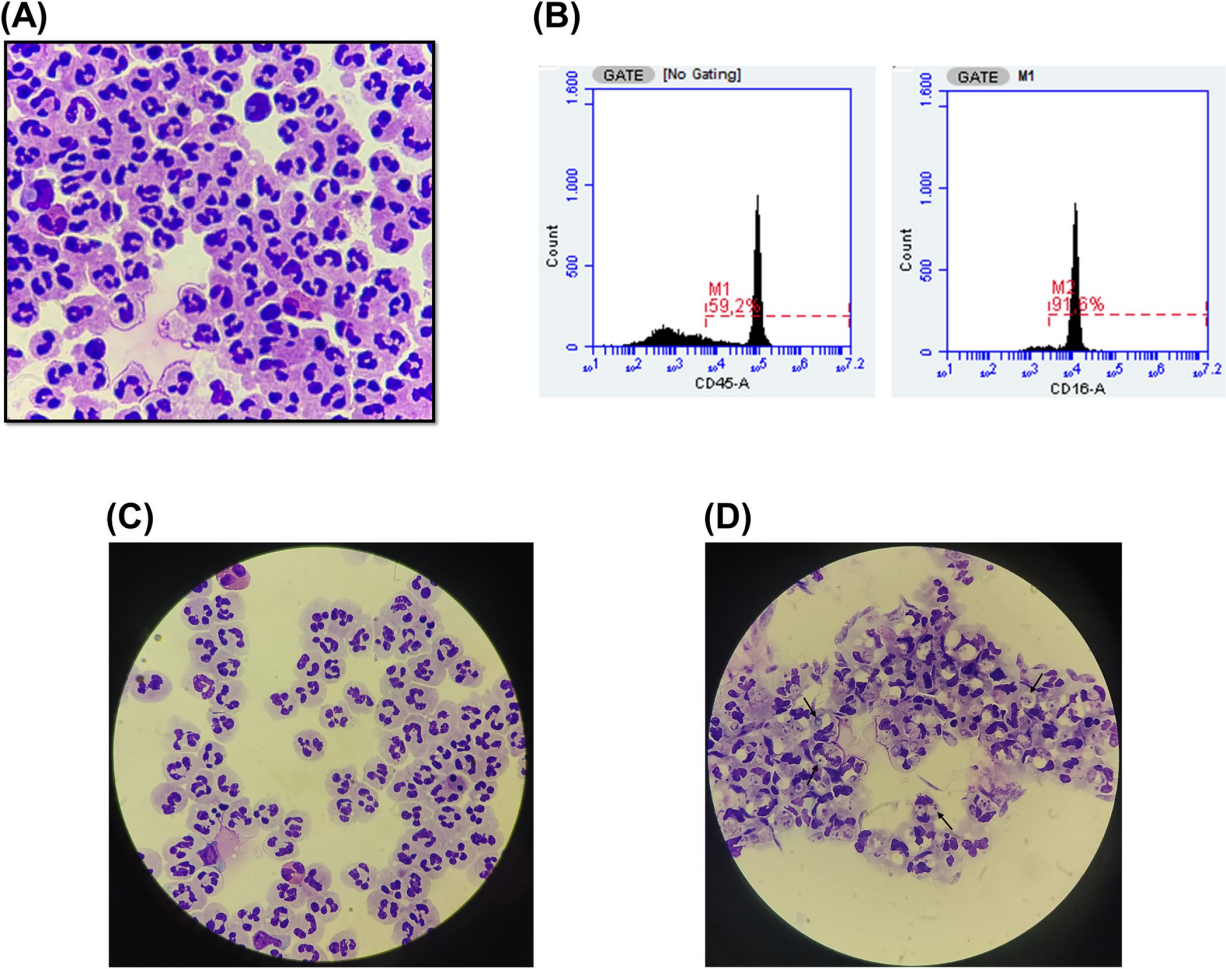
Neutrophils purified from whole blood of healthy male donors were infected with *L*. ***infantum*.** Representative image of (A) field of cytocentrifuge slide (100x magnification) comprised predominantly of neutrophils and (B) FACS of purified cells from sample 2, 59.2% were CD45+ leukocytes, thus analyzed for CD45+CD16+ resulting in 91,6% of neutrophils. Representative fields of cytocentrifuge slides (100x magnification) following a 3-hour culture period from (C) control samples comprising predominantly uninfected neutrophils and (D) neutrophils infected with the promastigote form of *L*. *infantum*. Black arrows indicate internalized *Leishmania* parasites, within parasitophorous vacuoles in neutrophils.

Neutrophils were isolated from whole blood of healthy male donors and its quantity and viability was evaluated using the Neubauer chamber with a Trypan Blue 0.4%. We found 20.26x10^6^±5.55 (mean±SD) neutrophils with a viability of 98.40%±1.14 (mean±SD). We also evaluated the purification process by microscopy of cytocentrifuge slides, observing the predominance of neutrophils (Fig 1A). After 3 hours, control samples presented a viability of 98.60%±1.14 did not differ from infected samples (95.60%±0.89). Cell surface markers CD45 and CD16 were also evaluated using flow cytometry, with 95.00%±2.24 double positive neutrophils (Fig 1B and S1 File).

Using a MOI 5:1, we observed a percentage of infected neutrophils of 89.80%±2.86 (mean±SD), with infected cells presenting 1.67±0.22 (mean±SD) *Leishmania* per infected cell. The infection index was 149.8±19.15 (mean±SD), showing that 3-hour exposure of neutrophils to *Leishmania* promastigotes was sufficient for infection of the cells (Fig 1C and 1D, and S2 File).

### Transcriptome profile is modulated during *in vitro* infection of human neutrophils by *L*. *infantum*

**Fig 2:**
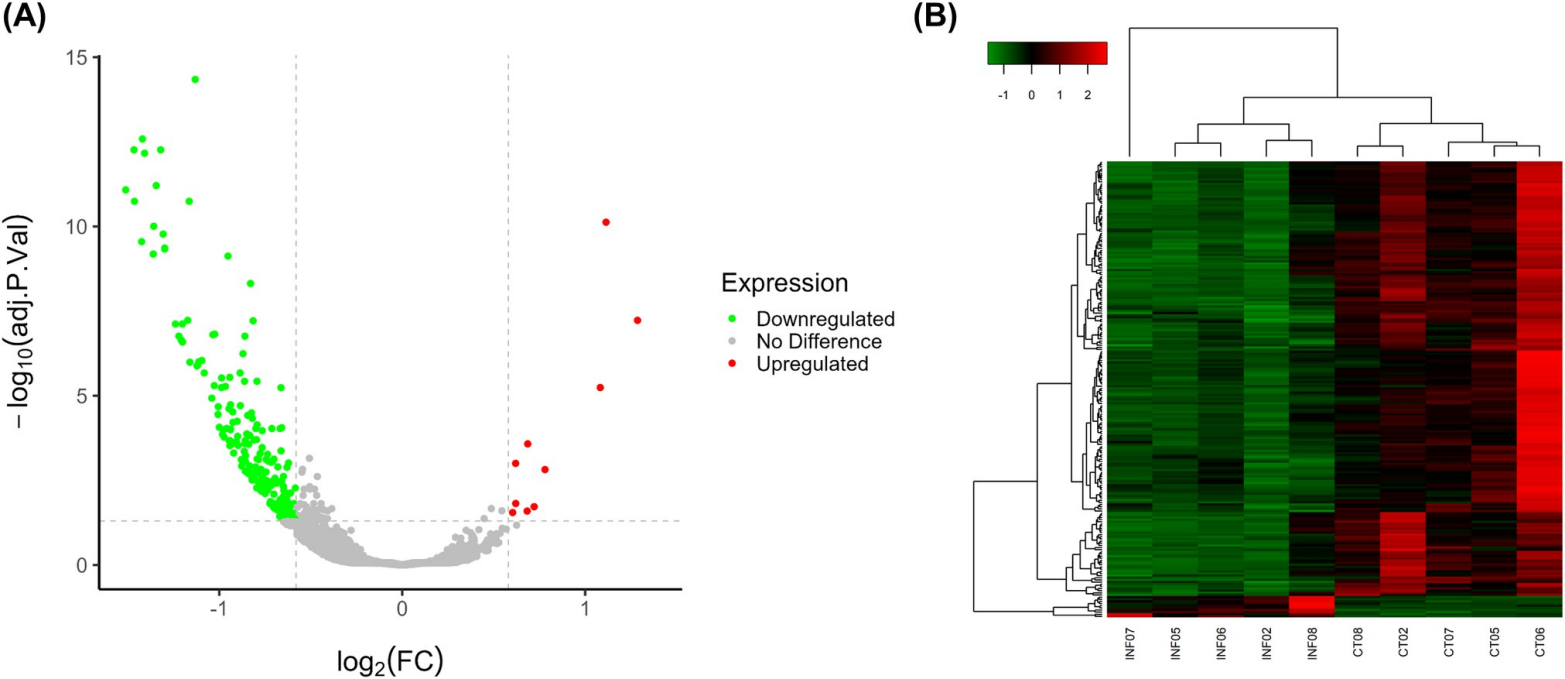
Transcripts modulated by *L*. *infantum* in human neutrophils. *In vitro* infection by *L*. *infantum* (INF) decreased expression of 202 transcripts and increased expression of 10 transcripts in human neutrophils when compared to control (CT). (A) Volcano plot shows 212 DEGs at log_2_(FC)±0.58 and FDR≤0.05 between human neutrophils infected and non-infected with *L*. *infantum*. Significantly upregulated transcripts (red dots) are in the upper right square of the graph (positive log_2_(FC) value) and significantly downregulated (green dots) are in the upper left square of the graph (negative log_2_(FC) value). (B) Heatmap shows normalized values for 212 differentially expressed transcripts (log_2_(FC)±0.58; FDR≤0.05). Transcripts were grouped by hierarchical clustering. This analysis was performed using heatmap3 package in RStudio and color scale represents z-score (green indicates lower expression, whereas red indicates higher expression).

Upon sample alignment with the human reference genome, control samples displayed an average alignment of 93.38% [93.1–93.9], whereas infected samples exhibited an average alignment of 69.14% [58.3–74.0]. Among the reads that failed to align with the human genome in infected samples, 82.14% [78.8–85.4] were in fact aligned with the *L*. *infantum* genome (*Leishmania infantum* JPCM5 Release 56, TriTrypDB), indicating a substantial presence of parasite RNA within our dataset. After exposure of neutrophils to *L*. *infantum*, we expected a change in the transcriptome of those cells, and our RNA-Seq analysis supported our hypothesis, revealing 212 DEGs (log_2_(FC)±0.58, FDR≤0.05) (S1 Table). According to BioMart annotations, 202 DEGs were classified as mRNA, 6 were classified as lncRNA and 4 had other classifications (S2 Table). Furthermore, 202 of the total transcripts were downregulated by infection with *L*. *infantum*, while 10 were upregulated in the same comparison (Fig 2).

Functional enrichment analysis resulted in 131 KEGG pathways, including different pathways related to immune response, mostly innate immune response (S3 Table). The pathway with the highest percentage of DEGs was Leishmaniasis, as can be seen in Table 1, which shows the top 10 KEGG pathways regulated by DEGs after infection of neutrophils with *L*. *infantum*.

**Table 1:** Pathway enrichment analysis for differentially expressed genes following *in vitro* infection of human neutrophils by *L*. *infantum*.

Pathway	% DEG	p-adj	DEG/Total	DEGs
Leishmaniasis	21.13	3.60E-21	15/71	*IFNGR1*, *CYBA*, *NCF4*, *NFKBIA*, *NFKB1*, *PTPN6*, *TLR4*, *TLR2*, *NCF1*, *IFNGR2*, *CYBB*, *ITGAM*, *MYD88*, *RELA*, *HLA-DRA*
Legionellosis	16.67	3.0E-12	09/54	*NFKB2*, *NFKBIA*, *NFKB1*, *TLR4*, *TLR2*, *ITGAM*, *CD14*, *MYD88*, *RELA*
Antigen processing and presentation	14.93	4.7E-13	10/67	*CTSS*, *CTSB*, *B2M*, *TAP1*, *HLA-DRA*, *HLA-C*, *HLA-E*, *HLA-F*, *TAPBP*, *HLA-B*
Allograft rejection	14.71	4.6E-07	05/34	*HLA-DRA*, *HLA-C*, *HLA-E*, *HLA-F*, *HLA-B*
PD-L1 expression and PD-1 checkpoint pathway in cancer	13.64	5.6E-15	12/88	*IFNGR1*, *HIF1A*, *NFKBIA*, *NFKB1*, *PTPN6*, *CD274*, *TLR4*, *TLR2*, *NFKBIE*, *IFNGR2*, *MYD88*, *RELA*
Graft-versus-host disease	13.51	6.8E-07	05/37	*HLA-DRA*, *HLA-C*, *HLA-E*, *HLA-F*, *HLA-B*
Phagosome	13.14	2.2E-21	18/137	*CYBA*, *NCF4*, *RAB5C*, *TLR4*, *TLR2*, *RAB5A*, *NCF1*, *CTSS*, *CYBB*, *TAP1*, *ITGAM*, *CD14*, *FCAR*, *HLA-DRA*, *HLA-C*, *HLA-E*, *HLA-F*, *HLA-B*
Ferroptosis	12.82	8.7E-07	05/39	*GCLC*, *HMOX1*, *CYBB*, *FTH1*, *NCOA4*
NF-kappa B signaling pathway	12.75	1.1E-15	13/102	*CFLAR*, *NFKB2*, *ICAM1*, *NFKBIA*, *NFKB1*, *TNFAIP3*, *TLR4*, *BCL2A1*, *CD14*, *MYD88*, *RELA*, *LYN*, *CCL4*
Viral myocarditis	12.73	4.9E-09	07/55	*BID*, *ICAM1*, *HLA-DRA*, *HLA-C*, *HLA-E*, *HLA-F*, *HLA-B*

Table 1 shows the top 10 pathways, organized according to the percentage of differentially expressed genes identified in the pathway, and contains the name of the pathway (Pathway), the percentage of differentially expressed genes (%DEGs), FDR value (p-adj) and ratio between identified DEGs in pathway and total number of genes in pathway, name of DEGs is also presented for each pathway.

### Microtranscriptome profile is modulated during *in vitro* infection of human neutrophils by *L*. *infantum*

From 6027 probes representing the human species in Affymetrix miRNA 4.1 Array strips, 446 were DE (FC±2, FDR≤0.01) in our experiment (S4 Table). Details regarding probe classification can be seen in S1 Fig.

**Fig 3:**
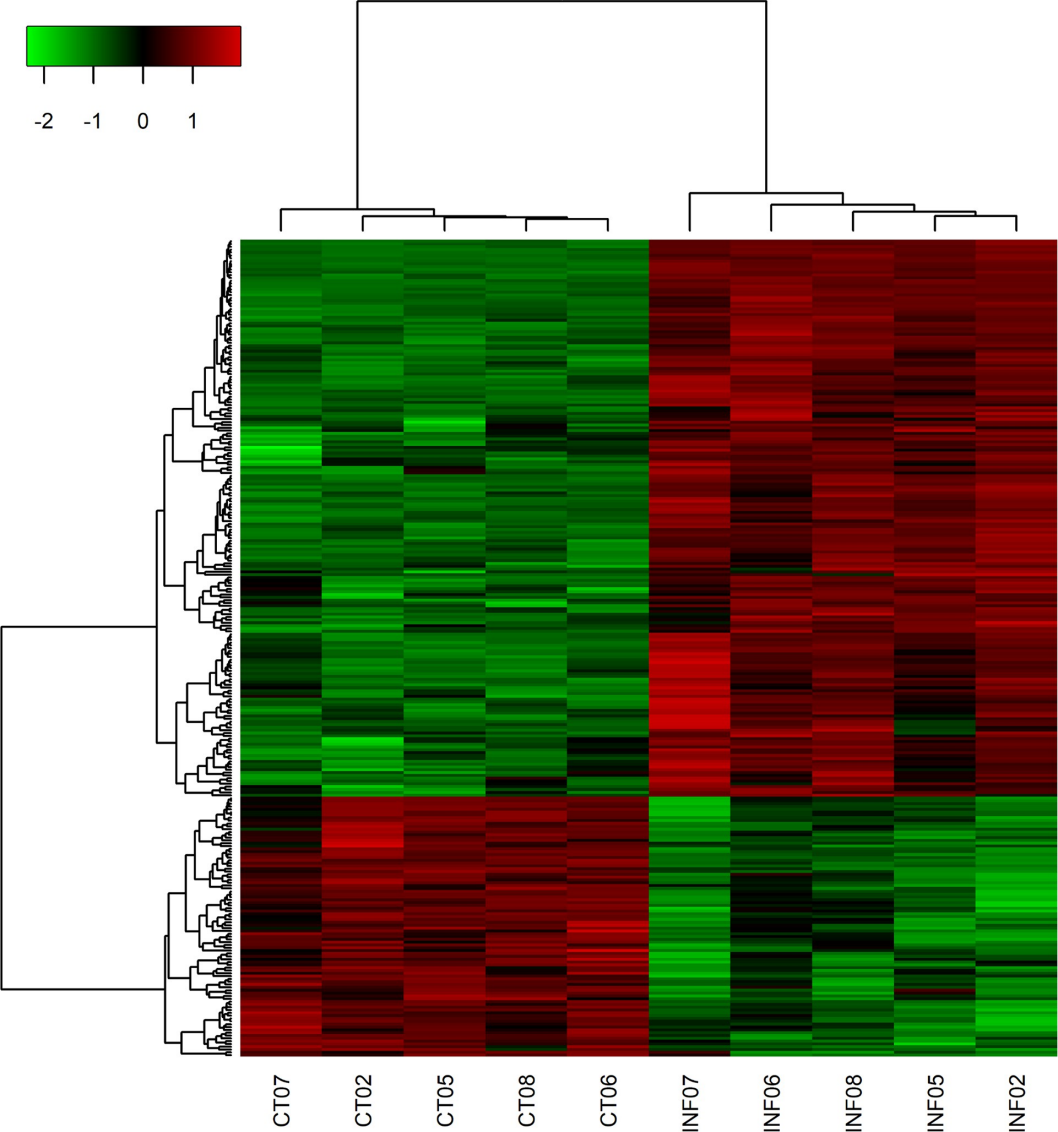
Heatmap of miRNAs modulated by *L*. *infantum* in human neutrophils. MiRNAs were grouped by hierarchical clustering. A total of 197 miRNAs were increased and 92 were decreased following *in vitro* infection of neutrophils with *L*. *infantum* (INF) when compared to control (CT). Heatmap shows corrected signal for 289 differentially expressed miRNAs (FC±2; FDR≤0.01). Analysis was performed using heatmap3 package in RStudio and color scale represents z-score (green indicates lower expression, whereas red indicates higher expression).

Our main interest with this experiment was to evaluate differential expression of mature miRNAs, since they can associate with seed region of expressed transcripts, preventing mRNA translation [48]. Thus, target prediction (see Methods) was carried out for 289 DE probes, representing mature miRNAs. From 289 miRNAs, 197 were increased in neutrophils post *L*. *infantum* infection, while 92 were decreased post-infection (p.i.) (Fig 3). In general, the action of miRNAs is inhibitory, impeding translation of mRNAs to proteins, so we considered as potential targets of upregulated miRNAs, all downregulated mRNA transcripts in the infected group of our RNA-Seq, whereas potential targets of downregulated miRNAs included the upregulated mRNAs in the same comparison.

**Fig 4:**
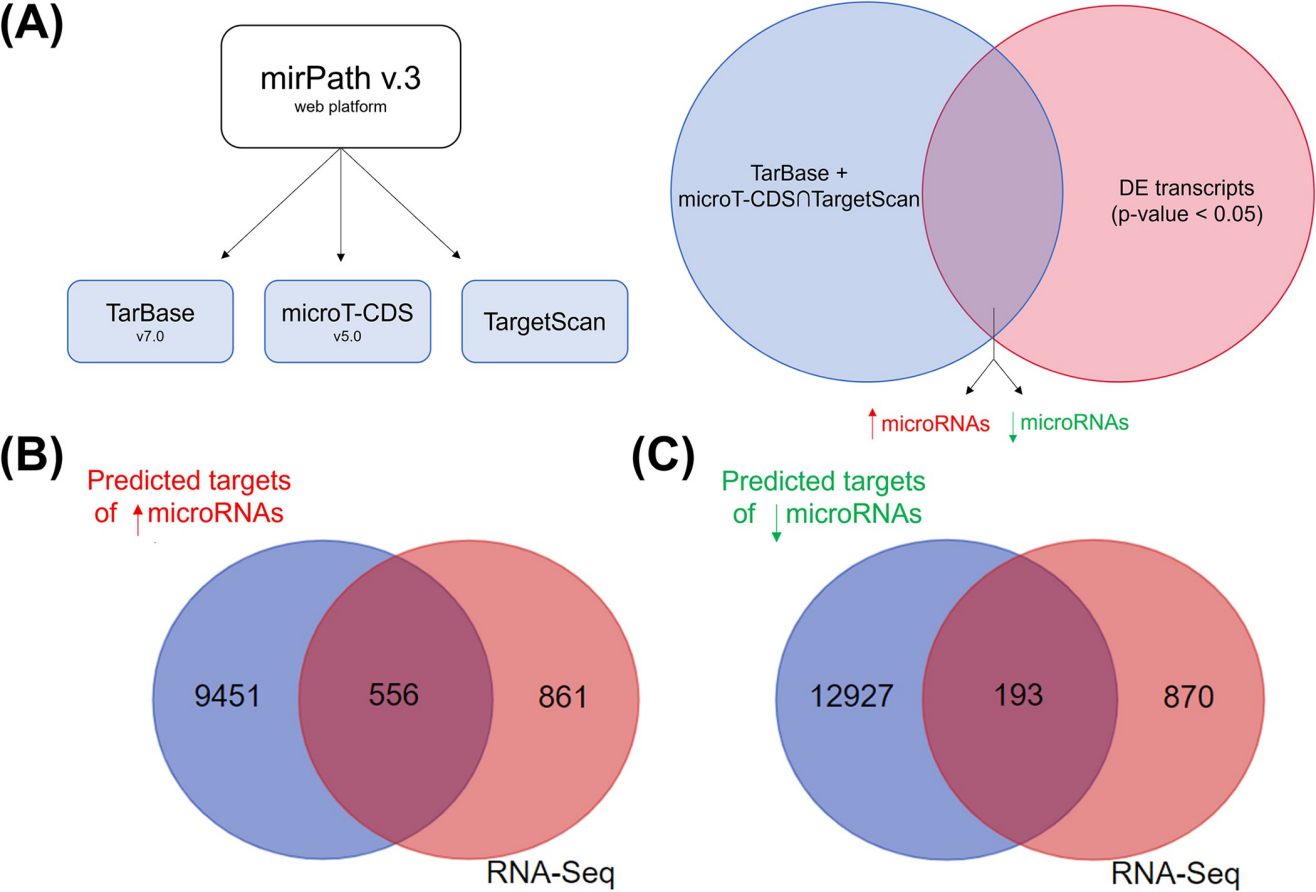
Target prediction of upregulated and downregulated miRNAs following *in vitro* infection of human neutrophils by *L*. *infantum*. (A) Differentially expressed miRNAs were divided according to expression patterns (up- or downregulated). DIANA-miRPath v3.0 (https://dianalab.e-ce.uth.gr/html/mirpathv3/index.php?r=mirpath) web platform was used to access three target prediction databases (TarBase, microT-CDS and TargetScan) for both, and then TarBase plus microT-CDS∩TargetScan predicted targets were compared to transcripts in our RNA-Seq analysis (p-value≤0.05). (B) 556 downregulated transcripts (p-value≤0.05) were found as potential targets of upregulated miRNAs. (C) 193 upregulated transcripts (p-value≤0.05) were found as potential targets of downregulated miRNAs.

For functional enrichment analysis, we compared targets predicted by TarBase, and added those predicted simultaneously in both, microT-CDS and TargetScan, with those DE coding transcripts in our data (Fig 4A). Thus, we found 556 downregulated transcripts, potential gene targets of the 197 upregulated miRNAs, while 193 upregulated transcripts have potential as targets for 92 downregulated miRNAs (Fig 4B and 4C).

### Changes in microtranscriptome profile following *L*. *infantum* infection of neutrophils affects immunological pathways

We analyzed up and downregulated miRNA target genes with the g:GOSt tool, from g:Profiler, resulting in functional enrichment analysis using KEGG database. For those downregulated transcripts in infected neutrophils, potential target genes of upregulated miRNAs, 181 pathways were enriched (FDR≤0.05), highlighting the Leishmaniasis Pathway, and several other inflammatory pathways such as Antigen processing and presentation, Toll-like receptor signaling pathway, NF-kappa B signaling pathway, JAK-STAT signaling pathway, among others. Top 10 pathways, organized according to the percentage of DEGs identified in the pathway, can be seen below (Table 2). Meanwhile, 29 pathways had FDR≤0.05 when using the upregulated transcripts in infected cells, potential targets of downregulated miRNAs. Among them, we predominantly observed the presence of physiological and constitutive cellular processes. All pathways regulated by both, upregulated and downregulated target transcripts, can be found in S5 Table.

**Table 2:** Pathway enrichment analysis for downregulated transcripts, targets of upregulated miRNAs, following *in vitro* infection of human neutrophils by *L*. *infantum*.

Pathway	% DEG	p-adj	DEG/Total	DEGs
Antigen processing and presentation	20.90	1.40E-14	14/67	*B2M*, *CD74*, *CTSS*, *HLA-A*, *HLA-B*, *HLA-C*, *HLA-E*, *HSPA5*, *PSME1*, *PSME2*, *RFX5*, *TAP1*, *TAP2*, *TAPBP*
Primary immunodeficiency	18.92	1.35E-07	07/37	*ADA*, *IL2RG*, *ORAI1*, *PTPRC*, *RFX5*, *TAP1*, *TAP2*
B cell receptor signaling pathway	18.42	7.93E-14	14/76	*GRB2*, *IKBKB*, *INPPL1*, *LILRB2*, *LILRB3*, *LYN*, *NFKB1*, *NFKBIA*, *NFKBIB*, *PIK3AP1*, *PIK3CD*, *RAC1*, *RAC2*, *RELA*
Viral myocarditis	18.18	3.67E-10	10/55	*ACTB*, *BID*, *EIF4G1*, *HLA-A*, *HLA-B*, *HLA-C*, *HLA-E*, *ICAM1*, *RAC1*, *RAC2*
Prolactin signaling pathway	17.91	6.73E-12	12/67	*CCND2*, *CISH*, *GRB2*, *IRF1*, *NFKB1*, *PIK3CD*, *RELA*, *SHC1*, *SOCS3*, *STAT1*, *STAT3*, *STAT5A*
Chronic myeloid leukemia	17.81	9.57E-13	13/73	*BCL2L1*, *E2F3*, *GAB2*, *GRB2*, *IKBKB*, *MDM2*, *NFKB1*, *NFKBIA*, *PIK3CD*, *RELA*, *SHC1*, *SMAD3*, *STAT5A*
Pancreatic cancer	17.57	1.12E-12	13/74	*BCL2L1*, *E2F3*, *IKBKB*, *NFKB1*, *PIK3CD*, *RAC1*, *RAC2*, *RALB*, *RALGDS*, *RELA*, *SMAD3*, *STAT1*, *STAT3*
PD-L1 expression and PD-1 checkpoint pathway in cancer	17.05	3.15E-14	15/88	*EML4*, *HIF1A*, *IFNGR1*, *IFNGR2*, *IKBKB*, *MAP2K3*, *MYD88*, *NFKB1*, *NFKBIA*, *NFKBIB*, *PIK3CD*, *RELA*, *STAT1*, *STAT3*, *TICAM1*
Fc gamma R-mediated phagocytosis	17.02	4.48E-15	16/94	*ACTR3*, *ARPC5*, *CFL1*, *GAB2*, *GSN*, *HCK*, *INPPL1*, *LIMK2*, *LYN*, *MARCKS*, *PIK3CD*, *PTPRC*, *RAC1*, *RAC2*, *VASP*, *WAS*
Leishmaniasis	16.90	1.33E-11	12/71	*C3*, *IFNGR1*, *IFNGR2*, *ITGAM*, *MYD88*, *NCF4*, *NFKB1*, *NFKBIA*, *NFKBIB*, *RELA*, *STAT1*, *TAB2*

Table 2 shows the top 10 pathways, organized according to the percentage of differentially expressed genes identified in the pathway, and contains the name of the pathway (Pathway), the percentage of differentially expressed genes (%DEGs), FDR value (p-adj) and ratio between identified DEGs in pathway and total number of genes in pathway, name of DEGs is also presented for each pathway.

### Long non-coding RNAs expressed in neutrophils, following *in vitro* infection with *L*. *infantum*, have *cis* and *trans* action

**Fig 5:**
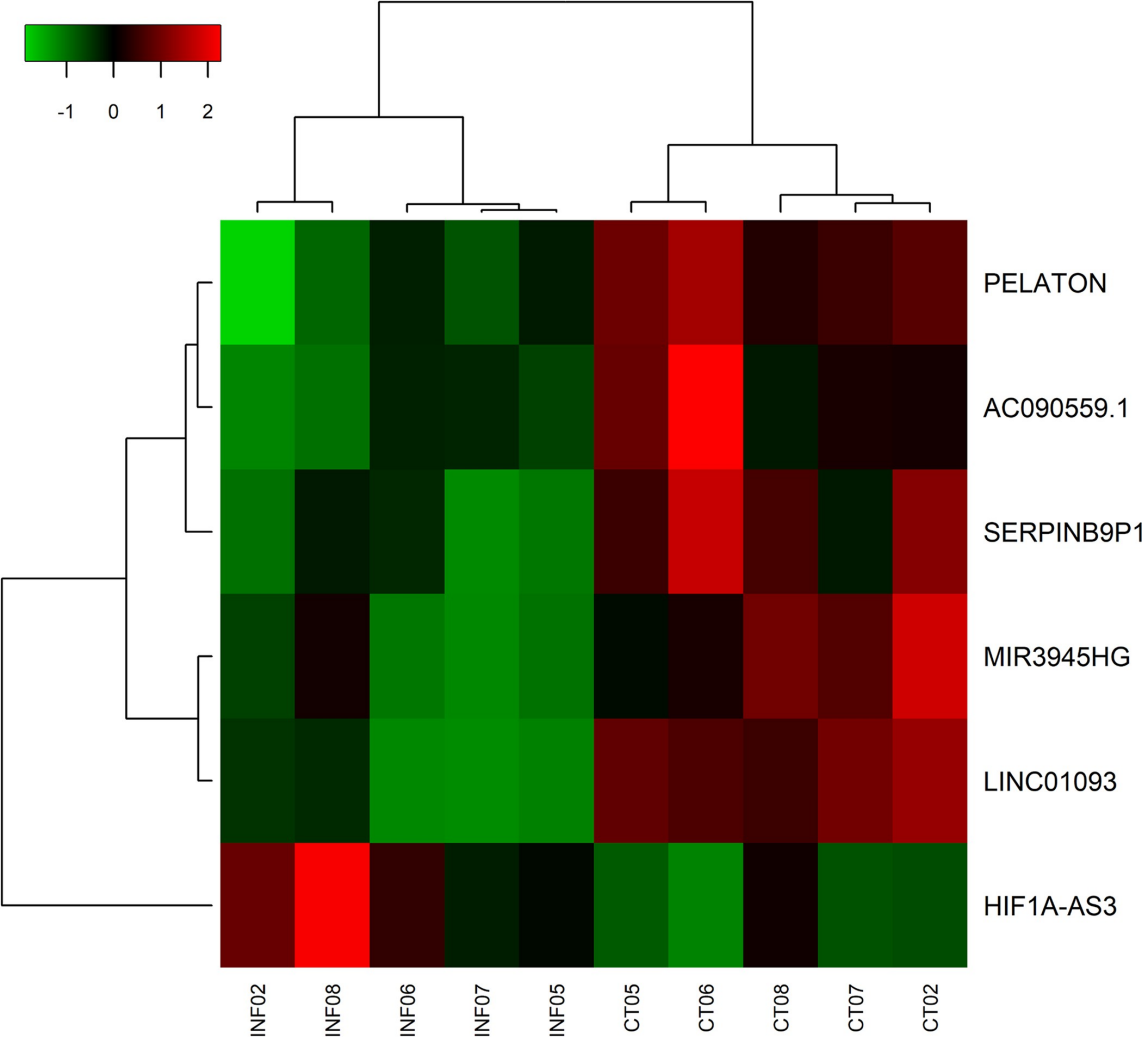
Heatmap of lncRNAs modulated by *L*. *infantum* following *in vitro* infection of human neutrophils. LncRNAs were grouped by hierarchical clustering. One lncRNA (HIF1A-AS3) was up regulated after *in vitro* infection with *L*. *infantum* (INF), while five (PELATON, AC090559.1, SERPINB9P1, MIR3945HG and LINC01093) were downregulated when compared to control (CT). Heatmap shows normalized values for six differentially expressed transcripts (log_2_(FC)±0.58; FDR≤0.05), classified as lncRNAs, using BioMart documentation (release 103). Analysis was performed using heatmap3 package in RStudio and color scale represents z-score (green indicates lower expression, whereas red indicates higher expression).

LncRNAs exert pre- and post-transcriptional regulation of gene expression [49]. After acute *in vitro* exposure of neutrophils to *L*. *infantum*, we observed a total of 6 DE lncRNAs (log_2_(FC)±0.58; FDR≤0.05). *HIF1A-AS3* was upregulated, while *PELATON*, *SLC39A13-AS1*, *SERPINB9P1*, *MIR3945HG*, and *LINC01093* were reduced in neutrophils p.i. (Fig 5).

LncRNAs can act as *cis* or *trans* regulators [reviewed [23]], therefore, to predict the action of these lncRNAs in our experimental model more accurately, we classified them in *cis* (lncRNA and target in chromosomal proximity) or *trans* (lncRNA and target distant from one another). After applying Pearson’s correlation on mRNA/lncRNA pairs (|r|≥0.8; p-value≤0.05), we observed 342 significant interactions. Pair *SPI1/SLC39A13-AS1* (r = 0.96; p-value<0.01) has been classified as *cis*-acting by the FEELnc algorithm. And 175 pairs, including all 6 lncRNAs DE, with ndG≤-0.10, according to the LncTar tool, were predicted to have a distant action (*trans*-targets) (S6 Table).

### Network analysis of DE mRNAs, DE lncRNAs and enriched pathways

**Fig 6:**
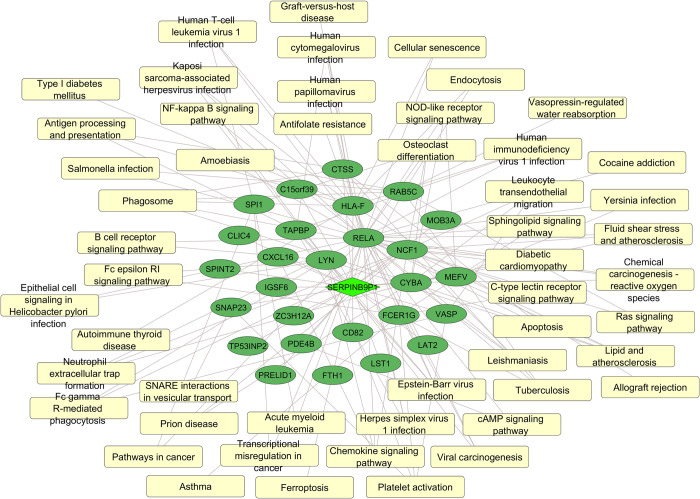
Network of co-expressed mRNAs/lncRNA and their enriched pathways, following *in vitro* infection of human neutrophils by *L*. *infantum*. LncRNA SERPINB9P1 and partner mRNAs, all positively correlated (|r|≥0.8; p-value≤0.05 and ndG≤-0.20) and downregulated after *in vitro* infection with *L*. *infantum* (green color), are shown. Pathways obtained after enrichment analysis of co-expressed transcripts are also represented. Diamond shape corresponds to lncRNA, ellipses correspond to mRNAs and rectangles to pathways. Analysis was performed using Cytoscape software (version 3.9.0).

For a broad view between co-expression of mRNAs/lncRNAs and their relationship with enriched pathways, we performed network analysis, using g:GOSt tool, from g:Profiler platform (https://biit.cs.ut.ee/gprofiler/gost) and Cytoscape software (version 3.9.0) (https://cytoscape.org/). The *trans*-acting *SERPINB9P1* lncRNA and its positively correlated (|r|≥0.8; p-value≤0.05) mRNAs had the highest binding potential (ndG≤-0.20) and is presented in Fig 6, highlighting once again the Leishmaniasis pathway and others closely related to immune response processes.

### Leishmaniasis pathway is regulated by mRNAs, miRNAs and lncRNAs

**Fig 7:**
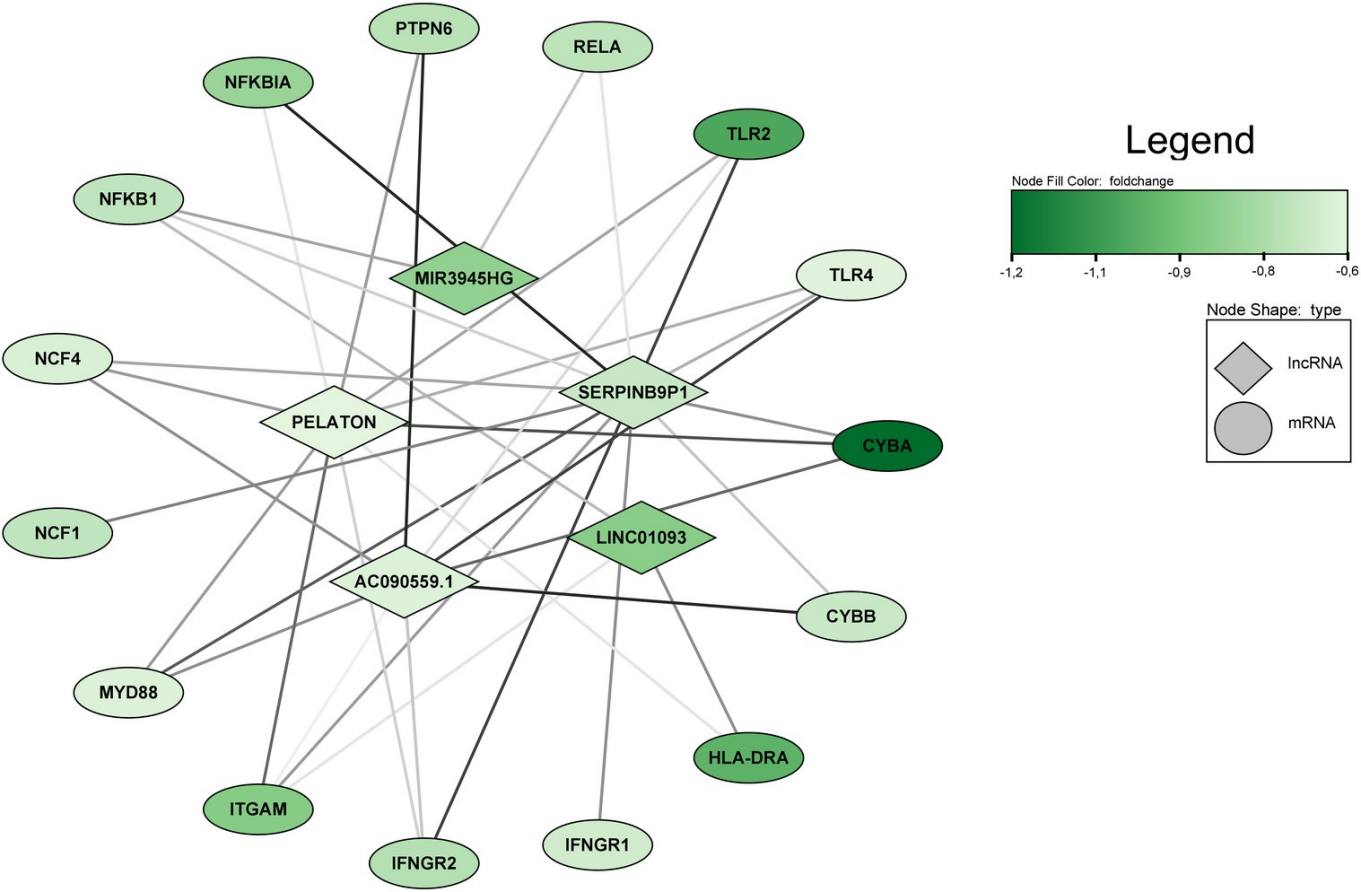
Network of co-expressed mRNAs/lncRNA present in Leishmaniasis Pathway. mRNA/lncRNA pairs, positively correlated (|r|≥0.8; p-value≤0.05 and ndG≤-0.10) enriching the Leishmaniasis pathway are presented, color of the lines varies according to correlation value. The higher |r|, darker the line. The color of the shapes varies according to log2fold change (FC) value. Darker green color represents lower expression. Diamond shape corresponds to lncRNA and ellipses correspond to mRNAs. Analysis was performed using Cytoscape software (version 3.9.0).

**Fig 8:**
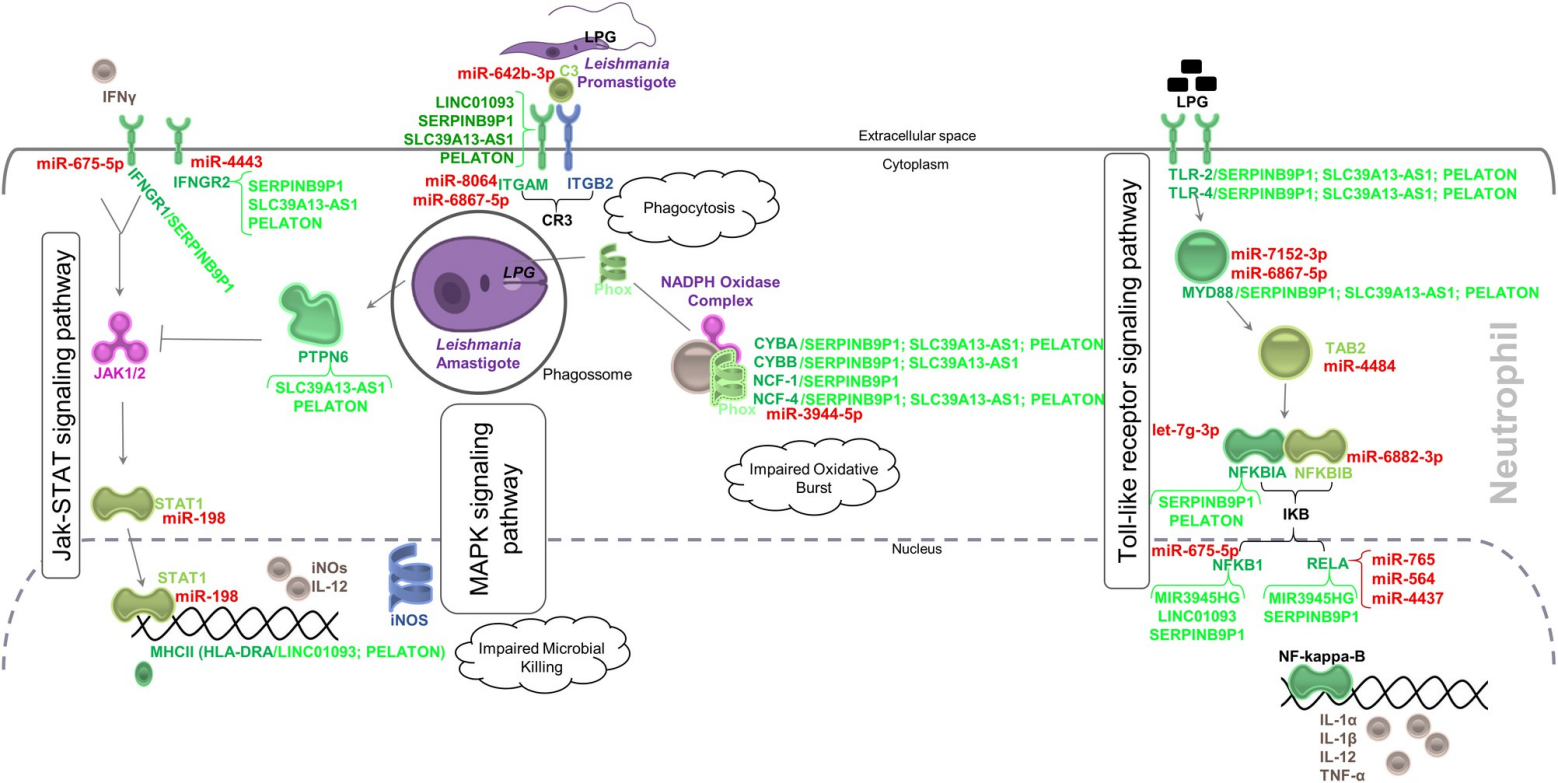
Regulation of the Leishmaniasis pathway following *in vitro* infection of human neutrophils by *L*. ***infantum*.** *L*. *infantum* infection impairs multiple immune signaling pathways, negatively affecting processes such as phagocytosis, apoptosis, and NO production, and can also impair pro-inflammatory signaling in human neutrophils. This suggests that *L*. *infantum* can regulate host response through increased expression of miRNAs. Green color represents downregulation of mRNA and correlated lncRNAs, red color indicates upregulation of miRNAs, molecule shape was based on the online tool https://targetexplorer.ingenuity.com/, and track functionality was based on KEGG database.

Of the 71 genes that compose the Leishmaniasis pathway, 15 DEGs downregulated (*CYBA*, *CYBB*, *HLA-DRA*, *IFNGR1*, *IFNGR2*, *ITGAM*, *MYD88*, *NCF1*, *NCF4*, *NFKB1*, *NFKBIA*, *PTPN6*, *RELA*, *TLR2*, and *TLR4*) (log_2_FC≤-0.58; FDR≤0.05) are lncRNA and/or miRNA targets, and 4 (*C3*, *NFKBIB*, *STAT1*, and *TAB2*) downregulated (p-value≤0.05) are miRNA targets, after *in vitro* infection of neutrophils by *L*. *infantum*. Of the 19, 12 are targets of p.i. upregulated miRNAs (Table 3). Also, DE mRNAs are positively correlated pairs of 5 DE lncRNAs (Fig 7). In summary, our DEGs regulate inflammatory signaling, phagocytosis and apoptosis in the Leishmaniasis pathway (Fig 8).

**Table 3:** Leishmaniasis pathway.

Pathway	% DEG	p-adj	DEG/Total	DEGs			miRNAs		
				Name	log2FC	p-adj	Name	FC	p-adj
Leishmaniasis	21.13	3.60E-21	15/71	C3	-0.61	5.34E-02	miR-642b-3p	10.08	1.27E-06
				CYBA	-1.20	7.59E-08	N/A		
				CYBB	-0.70	2.15E-02	N/A		
				HLA-DRA	-0.97	5.30E-06	N/A		
				IFNGR1	-0.68	2.37E-02	miR-675-5p	4.14	1.00E-04
				IFNGR2	-0.75	6.08E-03	miR-4443	10.34	1.27E-06
				ITGAM	-0.87	5.72E-07	miR-8064	3.64	8.80E-06
							miR-6867-5p	2.88	5.60E-03
				MYD88	-0.65	4.79E-03	miR-7152-3p	4.81	1.30E-03
							miR-6867-5p	2.88	5.60E-03
				NCF1	-0.72	1.53E-02	N/A		
				NCF4	-0.66	3.34E-03	miR-3944-5p	2.68	3.40E-03
				NFKB1	-0.72	4.34E-03	miR-675-5p	4.14	1.00E-04
				NFKBIA	-0.82	3.07E-03	let-7g-3p	3.78	4.90E-03
				NFKBIB	-0.35	3.41E-01	miR-6882-3p	2.63	3.60E-03
				PTPN6	-0.74	6.22E-03	N/A		
				RELA	-0.73	8.58E-04	miR-765	6.39	5.76E-05
							miR-564	3.98	1.50E-03
							miR-4437	3.42	6.00E-03
				STAT1	-0.33	4.80E-01	miR-198	5.75	3.00E-04
				TAB2	-0.44	2.32E-01	miR-4484	2.18	2.00E-03
				TLR2	-1.01	2.11E-05	N/A		
				TLR4	-0.64	2.01E-02	N/A		

Table 3 shows details of Leishmaniasis pathway, 15 differentially expressed genes (log_2_FC≤-0.58; FDR≤0.05) and 4 downregulated genes (p-value≤0.05), and 14 miRNAs (FC>2; FDR≤0.01) targeting 12 of them.

## Discussion

Our results have shown that 20 hours of exposure of human neutrophils to *L*. *infantum* leads to significant changes in the expression of coding and non-coding transcripts. These transcripts act in several immune related pathways, and the Leishmaniasis pathway was enriched with the highest percentage of DEGs (21.13%), all downregulated after *L*. *infantum* infection, yet more than half of DEGs are targets of p.i. upregulated miRNAs.

As part of the Leishmaniasis pathway, mRNA from both subunits of interferon-gamma (IFNγ) receptor were decreased in our neutrophils following *L*. *infantum* infection. Interestingly, Interferon Gamma Receptor 1 (*IFNGR1*) (log_2_(FC) = -0.68, FDR = 0.02) is a predicted target of the p.i. upregulated miR-675-5p (FC = 4.14, FDR<0.01) and is positively correlated to *SERPINB9P1* (log_2_(FC) = -0.70, FDR<0.01) lncRNA. *IFNGR1* orchestrates high-affinity, species-specific binding of ligands, while also regulating ligand trafficking and signal transduction. Similarly to our findings in neutrophils, *IFNGR1* expression is downregulated in human macrophages of patients affected by active VL (caused by *Leishmania donovani*) and in THP1 cells infected *in vitro* with *L*. *donovani*, and treatment with antimonial drugs was able to reverse this reduced expression in both cell types [50]. In addition, Interferon Gamma Receptor 2 (*IFNGR2*) (log_2_(FC) = -0.75, FDR<0.01) is a predicted target of the upregulated miR-4443 (FC = 10.34, FDR<0.01), besides being correlated to 3 lncRNAs (*SERPINB9P1*, *SLC39A13-AS1*, and *PELATON*). *IFNGR2* is also downregulated in monocyte-derived human macrophages following infection by *Leishmania major* [16]. In macrophages, recognition of IFNγ increases nitric oxide synthase (iNOS) production and therefore the production of nitric oxide, favoring the elimination of *Leishmania* amastigotes [51]. Likewise in our neutrophils, we propose that IFNγ signal transduction is impaired due to downregulation of its receptors, likely mediated by upregulated ncRNAs. Interference with Jak-STAT signaling pathway and decrease of *STAT1*, by upregulation of miR-198 (FC = 5.75, FDR<0.01), further supports a possible failure in transcriptional activation of iNOS, thus suggesting a possible impairment of NO production in infected neutrophils. Future functional studies are necessary to confirm our propositions.

An axis of the Leishmaniasis pathway mediates *Leishmania* phagocytosis and involves some of our DE transcripts, namely Complement receptor 3 (*CR3*) and the complement factor *C3* ligand. We observed *C3* downregulation p.i. (log_2_(FC) = -0.61, FDR = 0.05), and propose that this could be mediated by upregulation of miR-642b-3p (FC = 10.08, FDR<0.01). In mice, lack of *C3* complement impairs progression of skin lesions caused by *L*. *major*, decreasing the presence of neutrophils during infection [52]. CR3 is formed by two subunits: Integrin Subunit Alpha M (ITGAM), and Integrin Subunit Beta 2 (ITGB2). *ITGAM*, also called *CR3a*, is decreased p.i. in our experiment (log_2_(FC) = -0.87, FDR<0.01), and is a target of the upregulated miR-8064 and miR-6867-5p. This subunit is also positively correlated with *LINC01093* (log_2_(FC) = -0.86, FDR<0.01), *SERPINB9P1* (log_2_(FC) = -0.70, FDR<0.01), *SLC39A13-AS1* (log_2_(FC) = -0.65, FDR<0.05), and *PELATON* (log_2_(FC) = -0.63, FDR<0.01), and works as a neutrophil priming marker [53]. Neutrophil priming, first described by McPhail and Snyderman [54], regulates the intensity of neutrophil response at the site of inflammation [55] and enhances superoxide production. This process can occur in two ways: 1) rapid, with granule release or 2) delayed, activating transcription factors and stimulating cytokine production [55]. The increase of miR-8064 and miR-6867-5p could hinder phagocytosis of the parasites by neutrophils of the second wave of priming in neutrophils by decreasing *CR3a* following initial parasite internalization. After internalization of *Leishmania*, lipophosphoglycan present in phagocytosed promastigotes can interfere with NADPH Oxidase Complex formation by preventing *CYBA/CYBB* and *NCF1/NCF2/NCF4* binding, which leads to impaired oxidative burst and *Leishmania* resistance against host immune response [56]. Our results demonstrate that 4 components (*CYBA*, *CYBB*, *NCF1*, and *NCF4* mRNAs) of the NADPH Oxidase Complex were decreased following *L*. *infantum* infection. Thus, downregulation of these transcripts and an increase of miR-3944-5p, accounting for the decrease seen in its target, *NCF4* mRNA, could represent a mechanism of survival elicited by infection.

Within the Leishmaniasis pathway, members of the Toll-like receptor signaling pathway (*TLR2*, *TLR4*, *MYD88*, *NFKBIA*, *NFKB1*, and *RELA*) were all downregulated in neutrophils following *L*. *infantum* infection, and co-expressed with our 5 downregulated lncRNAs *(LINC01093*, *MIR3945HG*, *SERPINB9P1*, *SLC39A13-AS1*, and *PELATON*). Interestingly, we observe several upregulated miRNAs (let-7g-3p, miR-4437, miR-564, miR-675-5p, miR-6867-5p, miR-6882-3p, miR-7152-3p, and miR-765) predicted to target and, therefore, negatively regulate the aforementioned transcripts of the Toll-like receptor signaling pathway, thus impairing production of several pro-inflammatory cytokines, in our model. Effective killing of *Leishmania* and consequent control of infection appears to depend on correct downstream TLR signaling in other innate immune system cells [57–59]. In our experiment, members of the Toll-like receptor signaling pathway are targets of upregulated miRNAs, suggesting that the presence of the parasite affects immune response, at least partly, through modulation of miRNA expression in neutrophils.

We found 289 miRNAs DE after *L*. *infantum* infection in neutrophils, 197 up and 92 downregulated p.i.. Previously described as players in the pathogenicity of *Leishmania* [44], miRNAs seem to greatly influence the enriched pathways in our model. Functional enrichment of upregulated miRNA targets resulted in 181 pathways, predominantly related to immune response. We emphasize that 122 pathways are common to those enriched by all DEGs, despite being targets or not of miRNAs. The Leishmaniasis pathway, our focus herein, has 7% (14/197) of all upregulated miRNAs controlling the decrease of genes that participate in this pathway.

Increased expression for miRNAs following infection ranges from 2 to 261-fold. Among them, with the highest fold-change, miR-3167 had 19 predicted targets (microT-CDS∩TargetScan) and has never been reported in leishmaniasis. From all 19 predicted targets, only Metal Regulatory Transcription Factor 1 (*MTF1*) was DE (log_2_(FC) = -0.74; FDR<0.01) in our experiment. *MTF1* encodes a transcription factor that induces expression of metallothioneins, important low molecular weight proteins, responsible for carrying metals throughout the organism [60]. To date, only one study has associated *MTF1* to leishmaniasis, where knock-down of *MTF1* increases *L*.*V*. *panamensis* survival in THP-1 monocytes exposed to pentavalent antimonial drugs [61]. In macrophages, metallothioneins stimulate cytokine production [62], and since *MTF1* is required for their transcription, upregulation of miR-3167 could, in turn, negatively affect cytokine production.

Our top 5 miRNAs with the highest fold change are miR-3167, miR-4688, miR-7155-5p, miR-552-3p, and miR-642a-3p. So far, none of these miRNAs have been linked to *Leishmania* infection. But the enrichment of their 19 targets is related to glycosaminoglycan biosynthesis. In macrophages infected with *L*. *major*, degradation of glycosaminoglycans, and consequent carbon availability, is favorable for parasite survival. As our upregulated miRNAs seem to try to prevent the production of glycosaminoglycans, the unavailability of this molecule may be unfavorable to the presence of *Leishmania* parasites [63], indicating that our top 5 upregulated miRNAs act protectively against *Leishmania* infection.

In regards to the other type of ncRNA investigated in our study, lncRNAs are recently being studied in the context of *Leishmania* infection, these molecules with diverse regulatory functions seem to play a fundamental role in mounting immune response of the infected host [21,22]. In our neutrophil model, we found 6 lncRNAs DE, which represents less than 3% of DEGs, contrary to the findings by Fernandes *et al* who observed in macrophages infected with different species of *Leishmania* the presence of 24% of lncRNAs [22], a hypothesis for such a different finding would be, not only the use of different cells, but mainly the time of infection used, while the macrophages were exposed, among others, to *L*. *infantum* for 24 hours, our neutrophils were processed after 20 hours of exposure to the parasites.

We identified only one upregulated lncRNA–*HIF1A-AS3* (log_2_(FC) = 0.72, FDR = 0.02)–during acute *L*. *infantum* infection. This lncRNA was recently cited as the third identified antisense RNA of Hypoxia-Inducible Factor 1-Alpha (*HIF1A*) gene [64], which in turn is differentially downregulated in our study (log_2_(FC) = -1.36, FDR<0.01). We found no significant correlation between *HIF1A/HIF1A-AS3* pair. *HIF1A* gene limits the microbicidal ability of myelocyte-derived cells [65] and, in macrophages, *HIF1A* seems to play a fundamental role in survival of *L*. *donovani* at late phase (30 hours p.i.), but not at an earlier stage (6 hours p.i.) of infection [66]. Increase in *HIF1-AS3* appears to drive a positive feedback for the increase in *HIF1A* in human tumoral cells, through promoter transactivation [67]. Whether the *L*. *infantum* induced increase in *HIF1-AS3* lncRNA in our neutrophils could lead to an increase in *HIF1A* at later times, as part of the aforementioned positive feedback loop, remains to be investigated.

Understanding how the immune system of people with visceral leishmaniasis responds to this parasite is essential for the development of preventive and curative methods our study is the first to investigate the transcriptome and microtranscriptome of human neutrophils following *in vitro* infection with *L*. *infantum*. Observed changes in mRNA and ncRNA expression may impair phagocytosis, apoptosis and decrease nitric oxide production, favoring pathogen survival, and may decrease neutrophil priming in the first 20 hours of infection. We understand that without the use of inactivated parasites as a control, the general effects of phagocytosis on the transcriptome cannot be ruled out, and global expression changes reported herein must be followed by functional studies, to confirm pathophysiological changes.

Despite these limitations, our work sheds light on several mechanisms seemingly used by *L*. *infantum* to control neutrophil-mediated immune response, and identifies several promising targets (mRNAs, lncRNAs, and miRNAs) for targeted functional validation and association with neutrophilic function, aiming at the development of preventive or curative treatments for this prevalent zoonosis. We further suggest that research be carried out in purified peripheral blood human neutrophils from active VL patients compared to healthy individuals.

## Materials and methods

### Ethics statement

All procedures were approved by the local Research Ethics Committee—São Paulo State University (UNESP), School of Dentistry, Araçatuba (protocol number 3.926.267). Informed-consent documents were signed by all blood donors.

### Human subjects

Peripheral blood was collected from five healthy male donors (range 26–42 age) in vacuum tubes containing EDTA and in heparinized vacuum tubes. Blood samples collected in EDTA tubes were used to perform blood count for health assessment of each donor. All five donors had no history of recent infections, in addition to not showing any changes in their blood count (S3 File). To decrease confounding factors, we only collected samples from men, thus avoiding gender-specific hormonal variation.

### Maintenance of parasites

*L*. *infantum* promastigotes (MHOM/BR/00/MERO2) were isolated from a dog diagnosed with VL and further characterized by Sanger sequencing. Strain was maintained at 26°C in a complete Schneider medium (Sigma-Aldrich Co.), supplemented with 10% of inactivated fetal bovine serum (FBS), 2% sterile male urine and 1% of penicillin-streptomycin for 5–7 days, until they reach stationary phase, in addition, the passage of cultures did not exceed 3 times. Immediately before infection, promastigotes were tested for viability and absence of contamination and washed with phosphate buffered saline (PBS) (pH = 7.4) before infection of neutrophils.

### Neutrophil purification

Whole blood was immediately separated using a discontinuous density gradient [68] Histopaque-1119 (Sigma-Aldrich Co.). Briefly, heparinized blood was layered onto a double gradient Histopaque-1077/1119, and centrifuge at 700 × g for 30 minutes at room temperature, forming visible layers. Granulocyte layer was collected in a new tube, and any remnants red blood cells were hemolyzed with diluted (1:10) commercial solution Hemalise-RBC 10x (LGC Biotecnologia Ltda.). Cells were then PBS (pH = 7.4) washed, pelleted, and neutrophils were resuspended in commercial RPMI-1640/Hepes medium (LGC Biotecnologia Ltda.), supplemented with 10% of inactivated bovine fetal serum and 1% of penicillin-streptomycin.

Total number of neutrophils was counted in the Neubauer chamber and viability was estimated using Trypan Blue solution 0.4% (Life Technologies). In order to confirm purity, presumed neutrophils were cytocentrifuged and mounted on slides using a Cytospin centrifuge (Microprocessed Cytological Centrifuge, 2000 D, REVAN, Chientec) at 200 g for 5 minutes. Cytospin slides were stained using a commercial hematological staining (Panótico Rápido, Laborclin). The cells were examined by microscopy at 100x magnification.

### Flow cytometry analysis

To further assess the efficacy of neutrophil purification, flow cytometry was employed. Briefly, 1x10^5^ purified neutrophils were incubated with an Fc blocking buffer (10% FBS) for 30 minutes at room temperature. Purified neutrophils were centrifuged at 1800 rpm for 5 minutes and then incubated with Mouse Anti-Human CD45 conjugated to fluorescein isothiocyanate (FITC) (BD Biosciences, USA) and Mouse Anti-Human CD16 conjugated to phycoerythrin (PE) (BD Biosciences) in accordance to manufacturer’s instructions. Neutrophils displaying CD45/CD16 marks were subsequently analyzed. To avoid non-specific binding, cells were incubated with their respective control isotypes. All data acquisitions were counted in 10,000 events on channels FL1 and FL2, and cytometric analysis was performed using an Accuri C5 flow cytometer (BD Biosciences, USA) equipped with BD Accuri C6 software, version 1.0.264.21 (BD Biosciences, USA).

### *In vitro* infection

Neutrophils (10^6^/well) were added to 24-well plates, and those of infected group were exposed to promastigotes at a ratio of five *L*. *infantum* for each neutrophil (Multiplicity of Infection—MOI = 5:1). After 3 hours at 37°C and 5% CO_2_, neutrophils from the infected and control groups were washed with PBS (pH = 7.4)–in the infected group, PBS wash was used to remove non-internalized promastigotes, while in the control group same procedure was performed for sample pairing. At this point, cytocentrifugation technique was performed to confirm internalization of *L*. *infantum* in the infected group, counting at least 100 cells per sample, under microscope, for the quantification of the number of infected cells, as well as intracellular parasites within infected cells. Infection index was determined by multiplying the percentage of infected cells by the mean number of parasites per cell. Following the wash, neutrophils were resuspended in RPMI supplemented medium, and replated, maintaining the infected and control groups. After 20 hours total, viable neutrophils were washed and resuspended in PBS (pH = 7.4) for immediate total RNA extraction. Infection time was chosen based on the minimum time necessary for internalization of the parasite, as reported on previously published studies [69–71] while still maintaining high cell viability after 20 hours of total cell incubation.

### Total RNA extraction and quantification

Total RNA was extracted from 2x10^6^ neutrophils for each subject in both groups (n = 5 / group) using the miRVana kit (Thermo Fisher Scientific Co.) to preserve miRNAs, according to the manufacturer’s instructions. Immediately after extraction, samples concentration was measured on a NanoDrop 2000 spectrophotometer (Thermo Fisher Scientific Co.). Total RNA was kept at -80°C until further experiments were carried out.

### RNA Sequencing (RNA-Seq) and functional enrichment analysis of differentially expressed transcripts

Total RNA-Seq libraries were constructed from 150 ng of total RNA, using Zymo-Seq RiboFree Total RNA Library Kit (Zymo Research Co.), according to manufacturer’s instructions. Briefly, complementary DNA was synthesized using reverse transcription, ribosomal RNA was depleted, and adapters were attached before index library was amplified by Polymerase Chain Reaction. RNA-Seq libraries were sequenced on an Illumina NovaSeq sequencer to a sequencing depth of at least 30 million read pairs (150 bp paired-end sequencing) per sample, resulting in fastq files.

Quality control of raw reads (fastq files) was carried out using FastQC (Galaxy Version 0.73+galaxy0), available at the Galaxy web platform (www.usegalaxy.org). Illumina adapters were removed using Cutadapt (Galaxy Version 4.0+galaxy0) tool [72], and trimmed reads were aligned to human reference genome (assembly GRCh38.p13-v.34) with HISAT2 (Galaxy Version 2.2.1+galaxy0) [73]. Only reads exhibiting alignment to the human genome were taken into consideration for all subsequent procedures. Reads overlapping with exons were assigned to genes using FeatureCounts (Galaxy Version 2.0.1+galaxy2) [74]. Differential gene expression analysis was completed using DESeq2 (Galaxy Version 2.11.40.7+galaxy1) [75]. Genes with log_2_FoldChange (log2(FC))±0.58 and FDR≤0.05 were considered differentially expressed (DE).

Differentially expressed genes (DEGs) are subsequently used for functional enrichment analysis, performed using g:GOSt tool, from g:Profiler (version e108_eg55_p17_9f356ae) with Benjamini-Hochberg FDR multiple testing correction method applying significance threshold of 0.05.

### Microtranscriptome array, target prediction, and functional enrichment analysis

Total RNA (500 ng/sample) was labeled using the FlashTag Biotin HSR RNA Labeling Kit (Thermo Fisher Scientific Co.), according to the manufacturer’s instructions, including enzyme-linked oligosorbent assay as quality control. Hybridization to the Affymetrix miRNA 4.1 Array strips was carried out at 48°C for 20 hours. Subsequently, strips were processed and scanned using the GeneAtlas System (Affymetrix). Raw intensity values were background corrected, log_2_ transformed and then quantile normalized by the software Transcriptome Analysis Console (TAC) 4.0.1 (Thermo Fisher Scientific Co.) using the Robust Multi-Array Average (RMA) algorithm. Statistical analysis was also performed in the TAC software 4.0.1 (Thermo Fisher Scientific Co.) by one-way ANOVA, comparing infected *vs*. control group.

DE miRNAs (FC±2, FDR≤0.01) were separated into increased or decreased expression following *L*. *infantum* infection. All transcripts’ targets, available in the TarBase v7.0 database–a platform for previously experimentally observed targets–were considered [76]. Additionally, predicted transcripts in both microT-CDS v5.0 and TargetScan databases, simultaneously, were also considered [77,78]. DIANA-miRPath v3.0 (https://dianalab.e-ce.uth.gr/html/mirpathv3/index.php?r=mirpath) web platform was used to access all databases [79]. To improve our target prediction, we compared all predicted targets with our transcripts resulting from RNA-Seq analysis (p-value≤0.05), transcripts present in both were used as input for functional enrichment analysis with g:GOSt tool, from g:Profiler platform (version e108_eg55_p17_9f356ae; https://biit.cs.ut.ee/gprofiler/gost) applying the Benjamin-Hochberg FDR multiple testing correction method set at 0.05.

### Long non-coding RNA classification, mRNA/lncRNA co-expression, and network interaction

All DEGs (log_2_(FC)±0.58; FDR≤0.05) were submitted to biotype classification with the BioMart tool (release 103; www.ensembl.org/biomart/martview/) [80]. According to annotations relied on Ensembl’s coding/non-coding classification, transcripts biotyped as “lncRNA” were considered as lncRNAs, while those biotyped as “protein_coding” were considered as mRNAs, other classifications were not used for further analyses.

Pearson correlation coefficient was employed to calculate possible correlations between mRNA/lncRNA pairs (|r|≥0.8 and p-value≤0.05). Classifier Module of Flexible Extraction of Long Non-coding RNAs (FEELnc) tool [81] was utilized to predict nearby partner genes (those up to 100kbps distance), also called *cis*-targets. Further investigation with LncTar tool (http://www.cuilab.cn/lnctar), which estimates normalized free energy (ndG≤-0.10), was applied to predict the potential of interaction between lncRNAs and probable mRNA targets [82]; mRNA/lncRNA pairs with matching potential binding were assumed to be *trans*-targets, i.e., with distant action.

Gene co-expression networks were built using the Cytoscape software (version 3.9.0) (https://cytoscape.org/), with DE lncRNAs and their corresponding *cis* and *trans* target genes (|r|≥0.8; p-value≤0.05 and ndG≤-0.10). In order to contextualize the functional implications of gene alterations induced by *L*. *infantum* infection, we employed pathway enrichment analysis. g:GOSt tool, from g:Profiler platform (https://biit.cs.ut.ee/gprofiler/gost) was used to perform functional enrichment analysis using Kyoto Encyclopedia of Genes and Genomes (KEGG) pathways database, and also combined an integrated co-expression regulatory network.

## Supporting information

S1 Fig Probe distribution of small RNAs according to Affymetrix classification. Column plot represents distribution of total probes and differentially expressed probes contained in Affymetrix miRNA 4.1 Array strips. There are six possible classifications: mature microRNAs, stem-loop miRNAs, Small nucleolar RNAs, C/D box, H/ACA box, Small Cajal body-specific RNAs.
(TIF)

S1 File FACS analysis of CD45/CD16 double-positive neutrophils. (PDF)

S2 File Percentage of infected neutrophils, mean of amastigotes per infected cell, and infection index. (PDF)

S3 File Complete blood counts from all five male donors. (PDF)

S1 Table Differentially expressed genes (DEGs), resulting from RNA-Seq analyzes. A total of 212 DEGs (log_2_(FC)±0.58 and FDR≤0.05), being 202 with decreased expression after *L*. *infantum* infection, and 10 with increased expression post-infection.
(XLSX)

S2 Table Distribution of DE transcripts (RNA-Seq) according to ENSEMBL classification. DE transcripts (FDR≤0.05 and log_2_(FC)±0.58) were classified according to biotype in Biomart (ENSEMBL).
(XLSX)

S3 Table Pathways enriched by differentially expressed genes (DEGs). Differentially expressed genes (FDR≤0.05 and log_2_(FC)±0.58) were inserted on g:Profiler and resulted in 131 enriched pathways, most of them related to the immune system.
(XLSX)

S4 Table Differentially expressed miRNAs from microarray analyzes. A total of 446 probes were differentially expressed (FC±2 and FDR≤0.01) after *in vitro L*. *infantum* infection, 289 were classified as miRNAs.
(XLSX)

S5 Table Pathways enriched by up- and downregulated miRNA targets. (XLSX)

S6 Table Co-expressed mRNAs/lncRNAs pairs with binding potential. Pairs of mRNAs/lncRNAs that exhibited a Pearson correlation coefficient of |r|≥0.8 and p-value≤0.05 were evaluated for their binding potential in LncTar (ndG≤-0.10).
(XLSX)
