# Supplementary figures and images for: Leishmania infantum infection modulates messenger RNA, microRNA and long non-coding RNA expression in human neutrophils in vitro
Source: PLoS Negl Trop Dis. 2024 Jul 19;18(7):e0012318. doi: 10.1371/journal.pntd.0012318 (PMC11259272; doi:10.1371/journal.pntd.0012318)

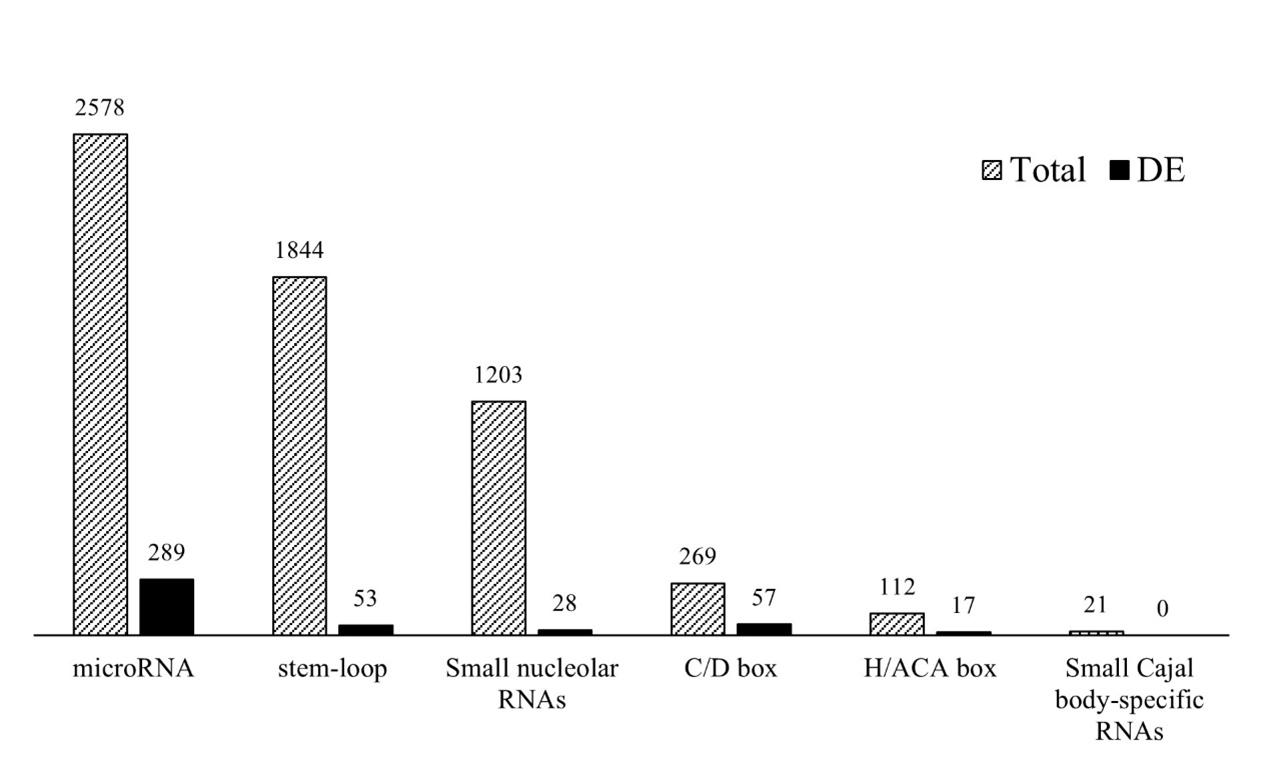

Supplement: S1 Fig — Column plot represents distribution of total probes and differentially expressed probes contained in Affymetrix miRNA 4.1 Array strips. There are six possible classifications: mature microRNAs, stem-loop miRNAs, Small nucleolar RNAs, C/D box, H/ACA box, Small Cajal body-specific RNAs. (TIF) [file pntd.0012318.s001.tif]
