# Supplementary material for: Leishmania infantum infection modulates messenger RNA, microRNA and long non-coding RNA expression in human neutrophils in vitro
Source: PLoS Negl Trop Dis. 2024 Jul 19;18(7):e0012318. doi: 10.1371/journal.pntd.0012318 (PMC11259272; doi:10.1371/journal.pntd.0012318)
Supplement: S1 File — (PDF) [file pntd.0012318.s002.pdf]

**Sample**	**$CD45^+CD16^+$**
2	91,64%
5	94,33%
6	97,18%
7	96,87%
8	94,96%
