# Supplementary material for: Leishmania infantum infection modulates messenger RNA, microRNA and long non-coding RNA expression in human neutrophils in vitro
Source: PLoS Negl Trop Dis. 2024 Jul 19;18(7):e0012318. doi: 10.1371/journal.pntd.0012318 (PMC11259272; doi:10.1371/journal.pntd.0012318)
Supplement: S2 File — (PDF) [file pntd.0012318.s003.pdf]

SAMPLE	Percentage of infected neutrophils
INF02	86%
INF05	88%
INF06	90%
INF07	92%
INF08	93%

SAMPLE	Mean number of parasites per infected neutrophil cell
INF02	1.84
INF05	1.74
INF06	1.47
INF07	1.41
INF08	1.89

SAMPLE	Infection index
INF02	158.24
INF05	153.12
INF06	132.30
INF07	129.72
INF08	175.77
