# Supplementary material for: Leishmania infantum infection modulates messenger RNA, microRNA and long non-coding RNA expression in human neutrophils in vitro
Source: PLoS Negl Trop Dis. 2024 Jul 19;18(7):e0012318. doi: 10.1371/journal.pntd.0012318 (PMC11259272; doi:10.1371/journal.pntd.0012318)
Supplement: S3 File — (PDF) [file pntd.0012318.s004.pdf]

BLOOD COUNT						
Samples						Reference values
	2	5	6	7	8	
Age (years)	26	26	42	28	32	
Red blood cells (millions/mm³)	5.05	4.9	5.45	4.62	4.88	4.50 - 5.90 millions/mm³
Hemoglobin (g %)	15.5	15.1	16	13.9	14.6	13.50 - 17.50 g %
Hematocrit (%)	46	46	47.5	42	44.4	41.00 - 53.00 %
Average corpuscular volume (µm³)	91.09	93.88	87.16	90.91	90.98	80.00 - 100.00 µm³
Average corpuscular hemoglobin (pg)	30.69	30.82	29.36	30.09	29.92	25.00 - 34.00 pg
Average corpuscular hemoglobin concentration (g/dL)	33.7	32.83	33.68	33.1	32.88	31.00 - 36.00 g/dL
Red cell distribution range (RDW) (%)	12.2	12.2	12.4	12.2	12	11.50 - 15.00 %
Platelets (mm³)	259,000	217,000	229,000	249,000	245,000	150,000 - 450,000 mm³
White blood cells (mm³)	7,460	6,110	5,050	3,990	7,020	3,500 - 10,500 mm³
Absolute band neutrophils (mm³)	0	0	50	0	0	0 - 525 mm³
Absolute neutrophils (mm³)	3,954	2,994	2,272	1,716	3,861	1,575 - 7,350 mm³
Absolute eosinophils (mm³)	373	122	252	120	281	35 - 525 mm³
Absolute basophils (mm³)	0	61	0	0	0	0 - 105 mm³
Absolute lymphocytes (mm³)	2,835	2,505	2,272	1,875	2,246	700 - 4,725 mm³
Absolute monocytes (mm³)	298	428	202	279	632	70 - 1,050 mm³
Method: Automation BC-5380 Mindray						
